# Multiple microvessels extending from the coronary arteries to the left ventricle in a middle aged female presenting with ischaemic chest pain: a case report

**DOI:** 10.1186/1752-1947-1-177

**Published:** 2007-12-10

**Authors:** Robert J MacFadyen, Chetan Varma, Robert H Anderson

**Affiliations:** 1 University Department of Medicine and Department of Cardiology, City Hospital, Dudley Road, Birmingham B18 7QH, UK; 2 Cardiac Unit, Institute of Child Health, University College, London, UK

## Abstract

Possible ischaemic chest pain presentations are exceedingly common. Angiographic triage of clinical, electrocardiographic or biomarker positive presentations is increasingly feasible with the expansion of cardiac catheterization facilities. This management pattern often extends to problem patients with negative biomarker screens whose symptoms appear unstable. With invasive triage even very rare congenital or developmental coronary anomalies will be more frequently recognized although their relationship to ischaemia can be confounded by association. In this a case we report a woman with widespread direct coro-ventricular micro-channel formation across the heart and an ischaemic presentation, despite angiographically normal epicoronary vessels. This pattern, while very rare, needs to be recognized as one possible phenotype in this very common clinical presentation.

## Introduction

Congenital variants in the structure or positioning of the native coronary arteries, or acquired coronary-cameral fistulas in the adult, are rare but well documented [1]. They are often defined in routine diagnostic and/or therapeutic coronary angiographic procedures following related or unrelated symptomatic presentation. Some may be linked to symptomatic ischaemia, with or without conventional atherosclerotic coronary arterial stenoses. In contrast to single arterio-venous or arterio-arterial fistulas, direct microfistulas between individual coronary arteries and the left ventricle are exceedingly rare [2]. We report a patient with multiple micro-channels extending from both coronary arteries to the left ventricle, who presented with acute chest pain typical of myocardial ischaemia.

## Case presentation

The patient (Caucasian; female; 58 yr; 83 kg; BMI 32; para 2^+0^) initially presented to a district hospital with an abrupt history of typically ischaemic exertional chest pain. The pain occurred with a stable frequency, but had increased in severity over several months. Her general and cardiac examination was unremarkable, with the exception of community-based treatment for hypertension. Her presenting 12 lead ECG, and repeated measurements of cardiac troponin I, showed no abnormality. She had been discharged from the admitting hospital for elective cardiac investigation, but was re-admitted within 48 hours because of symptoms of recurrent pain, along with the concerns of both the patient and her general medical practitioner. On the repeat admission, there were again no changes in her repeat ECG and cardiac biomarkers, including troponin I, which were persistently negative. She had had no symptomatic response to additional oral anti-ischaemic therapy (Bisoprolol). Due to persistent symptomatic problems, and a suspicion of reversible ischaemia despite negative biomarkers and normal ECG and chest x-ray, she was transferred for emergent angiographic triage and/or percutaneous coronary intervention. No functional testing had been completed prior to transfer due to the patient's age and gender and given that non invasive tests have well documented poor specificity and sensitivity in female hypertensive patients in midlife [3].

The patient was noted to be asymptomatic on transfer, and her chronic therapies had been adjusted to treatment with Bisoprolol 5 mg od, Aspirin 75 mg od; Simvastatin 40 mg on and Amlodipine 5 mg od. Her accompanying chest x-ray showed a normal cardiac silhouette later confirmed by transthoracic echocardiography showing no abnormality of valvular structure or contractile function. The following day, she was taken to diagnostic cardiac catheterization, with a view to proceeding to percutaneous intervention if required.

**Figure 1:**
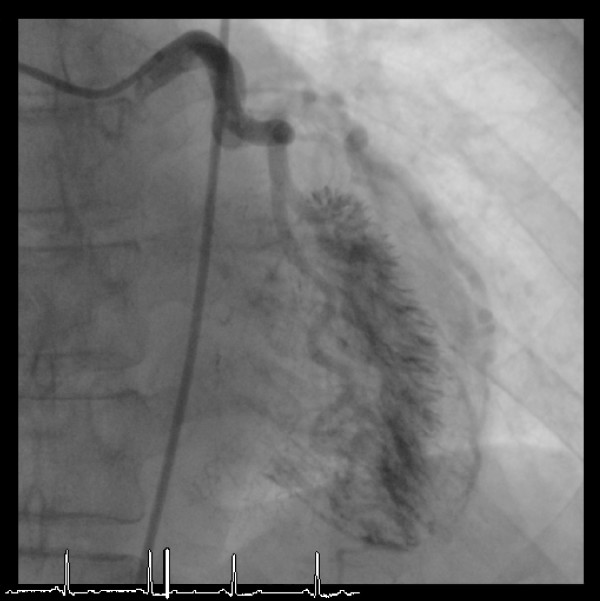
Late intra-coronary injection of contrast in the straight antero-posterior view shows multiple transmural micro-channels emptying directly into the left ventricle.

**Figure 2:**
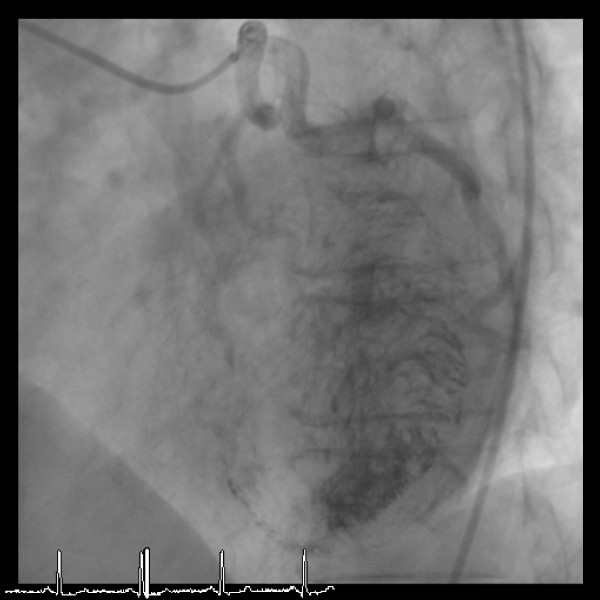
The late intra-coronary injection, when viewed in the LAO cranial projection, shows that the micro-channels extend from all anterior coronary arterial branches to fill the pre-systolic left ventricle.

**Figure 3:**
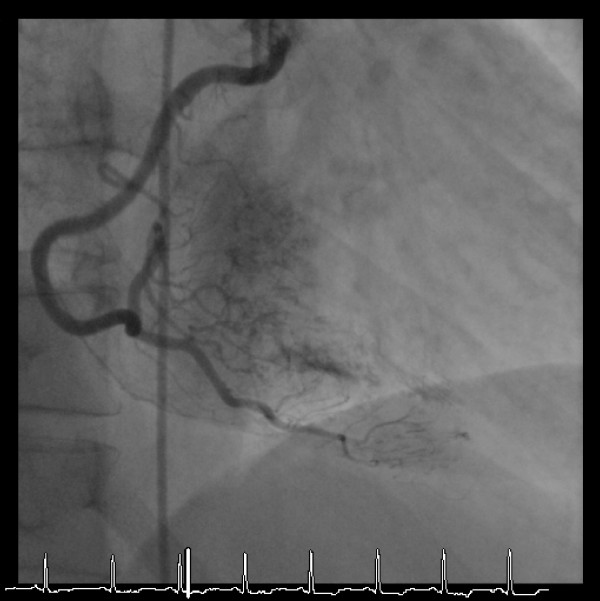
A late intra-coronary injection of contrast visualized in the straight LAO projection shows intra-muscular micro-channels also extending from the right coronary artery to fill the left ventricle.

Routine selective coronary angiography was completed uneventfully from the right femoral artery, using lignocaine local anaesthesia. Initial injections into the left coronary artery revealed diffuse direct shunting of contrast from all branches into the left ventricular cavity throughout the length of the vessel (Figures 1 and 2). The epicardial coronary arterial tree was otherwise normal, showing no trace of atheroma, and no evidence of isolated arterial stenosis. Spontaneous coronary spasm was not documented. Conventional imaging failed to visualize the entire length of the communications joining the coronary arteries to the cavity of the left ventricle. The right coronary artery was also affected by the same phenomenon, but to a more minor degree, with the micro-shunting occurring predominantly distally and towards the apex of the heart (Figure 3). The contrast left ventriculogram was normal.

The patient was reassured with these findings, and treated symptomatically by continued use of vasodilators for blood pressure control. She remained well and asymptomatic at follow-up when reviewed through to 18 months after the index admission. An outpatient myocardial adenosine stress scintigram was completed. This showed normal rest and stress myocardial perfusion. Following this the patient was discharged from regular hospital review.

## Discussion

Acquired and/or congenital coronary-cameral fistulas are rare, but well documented. In most instances, they take the form of isolated vessels of large caliber. They generally pass directly into the chambers of the heart from the proximal right or left coronary arteries [4], but can also form channels to related organs within the thorax, such as the pulmonary or bronchial circulations [5]. Multiple micro-fistulas passing blood directly into the cavity of the left ventricle tend to be distal, and are a very rare phenomenon. The mechanism of formation of these channels and their functional impact is less clear. They may be structurally distinct from more frequently seen isolated coronary-cameral, coronary-visceral, or coronary arterio-venous fistulas [6].

The majority of the published examples of these abnormalities are as individual case studies, which due to their rarity, have appeared sporadically over the last 25 years [7]. Said and van der Werf, nonetheless, recently gathered information on 20 cases collated from across the Netherlands [2]. Multiple micro-vessels extending from the coronary arteries and feeding multiple coronary territories (as in this case) were found in only one patient. In their experience, female gender was common, and the majority of their patients, as in our case, are in mid life or older, with no demonstrable epicardial atherosclerotic coronary arterial disease. These patients have a predictable high prevalence of associated presentation with chest pain symptoms leading to angiographic triage. The linkage to female gender may therefore be confounded by association and well known difficulties in non invasive triage of female patients in midlife particularly where there are concomitant risk factors for coronary disease (such as hypertension). Structural and electrocardiographic abnormalities, such as ventricular hypertrophy, are common although neither was seen in our patient.

The relationship of these congenital micro channels to emergent infarction or recurrent true ischaemia remains unclear. It has been suggested that these patients may account for a small proportion of cases of myocardial infarction without atherosclerotic coronary arterial disease, although again the mechanisms for this are unclear and anatomical cases are exceedingly rare. Longer term management of this circulation is problematic. Clearly surgical ligation is not feasible, although it has occasionally been used with success when isolated fistulas have been shown to cause arterial steal and demonstrable ischaemia. Our patient who went on to demonstrate normal myocardial perfusion was managed empirically with multiple vasodilator therapy, with good effect. Given the variable arterial structure of these channels and their intramural location (exposed therefore to the pulsatile flow within the cardiac cycle), the rationale or longer term efficacy for this regimen is uncertain.

## Conclusion

Patients with multiple micro-channels extending from the coronary arteries to the ventricular cavities can be seen rarely in midlife in females presenting with troponin-negative ischaemic chest pain. Given the increasingly common pattern of invasive triage despite negative biomarkers, and the possible non invasive definition of these anomalies, the true prevalence of these cases has yet to emerge. Longer term management continues to be based empirically on relief of presumed ischaemia even where this is not demonstrable on functional testing. As in our patient, vasodilator therapy can at least be symptomatically effective.

## Competing interests

The author(s) declare that they have no competing interests.

## Authors' contributions

RJM completed the diagnostic cardiac catheterization, drafted and revised the manuscript, obtained consent from the patient, contributed to patient supervision and completed the clinical follow up. CV acted as the initial admitting Consultant triaging for investigation and contributed revisions to the draft manuscript. RHA commented on the case, contributed to revisions of the manuscript draft and acted as an independent advisor on cardiac anatomy. All authors read and approved the final manuscript.

## Consent

Our patient reported here gave her written informed consent to the anonymous description of her case presentation in this publication.
